# Impact of Social Media on Choosing Skin Care and Cosmetic Products Among Females in Saudi Arabia

**DOI:** 10.7759/cureus.49922

**Published:** 2023-12-04

**Authors:** Maryam A Alamer, Hatim Alrashed, Bandar M Abuageelah, Lina I Kinkar, Zahraa A Alwayel, Mona H Alfaifi, Mahdi T Alfataih, Leena M Alzakry, Ziyad M Alruwaili, Aminah A Alhumam

**Affiliations:** 1 Medicine and Surgery, College of Medicine, Jazan University, Jazan, SAU; 2 Medicine and Surgery, College of Medicine, Jouf University, Sakaka, SAU; 3 Medicine and Surgery, Batterjee Medical College, Aseer, SAU; 4 Medicine and Surgery, College of Medicine, Umm Al-Qura University, Makkah, SAU; 5 Medicine and Surgery, College of Medicine, King Faisal University, Alahsaa, SAU; 6 Medicine and Surgery, College of Medicine, Najran University, Najran, SAU; 7 Medicine and Surgery, College of Medicine, Imam Mohammed Ibn Saud Islamic University, Riyadh, SAU; 8 Dermatology, College of Medicine, King Faisal University, Alahsaa, SAU

**Keywords:** saudi arabia, females, impact, use, skin care products, cosmetics, social media

## Abstract

Background: Social media is a network that allows information to be shared globally with millions of users. It is becoming evident that social media plays quite a prominent role these days in skincare. Social media surely has come to benefit millions of its users around the globe, but the downside of social media is that it has the potential to put users at risk while they follow popular trends.

Aim: This study aims to assess the impact of social media on choosing skincare and cosmetic products in Saudi Arabia with the most used social media platforms.

Methods: A questionnaire-based cross-sectional study was conducted targeting adult female residents across Saudi Arabia. Data were collected from the participants who met our criteria via electronic data collection Google forms did not show any nominative information that was distributed through social media platforms. The questionnaire covered participants' demographic data, social media use, source of information, and degree of trust with the influence of social media on using cosmetics. The eligible females were asked to fill out the study questionnaire received till no more new answers were obtained.

Results: A total of 1,174 females fulfilling the inclusion criteria completed the study questionnaire. Participants' ages ranged from 18 to more than 40 years with a mean age of 22.5 %C2%B1 13.9 years old. Exact of 655 (55.8%) were single, and 463 (39.4%) were married. The most used social media platforms included Snapchat (39.4%), TikTok (26.7%), and Instagram (19.6%). A total of 881 (75%) of the study females reported they use social media for more than an hour a day. Exact 51% of the study females became familiar with skin care products from social media platforms. Also, 91.3% of the study female's confidence in information related to cosmetic and skin care products was affected by visual presentation.

Conclusion: In conclusion, the study showed that most of the study participants used social media for many hours daily mainly Snapchat, TikTok, and Instagram. Also, social media was the main source of information regarding skin care products mainly dermatologists on social media.

## Introduction

Unlike traditional media outlets, social media uses two-way communication technologies that enable users to begin online dialogues, express questions, remarks, or both, and get a response [1]. Social networking platforms are becoming more popular among friends. As a result, it is another compelling reason for businesses to engage with their customers and market their products through social media [1,2]. Lately, social media usage in healthcare has recognized many implications of patients using social media for healthcare-related reasons within the healthcare system, and sometimes it can aid patients [3,4]. About 74% of college students search for healthcare-related information on the Internet [5]. Social media has undeniably helped millions of people globally. However, the downside of social media is that it may put individuals in danger when they follow popular patterns. Following skincare trends without knowing their impact on one's skin could be hazardous. Skincare and beauty influencers have a significant effect on audience product buying choices [6,7]. As for aesthetic medicine, numerous people are highly convinced by the information they see on the social media platforms of many influencers and cosmetic/dermatologist doctors [8]. Furthermore, a new study revealed that 41% of patients follow their current or potential provider on Instagram, and the presence of providers on social media influenced 43% of customers’ decisions to make an appointment. Also noted is that social media ranked among the top three factors to consider when purchasing skin care products and sixth for whether to have a cosmetic treatment [9-11]. This study will be conducted to evaluate the impact of social media on choosing skincare and cosmetic products in Saudi Arabia, to assess the most used and most popular social media platform and its massive effect on choosing skincare and cosmetic products, and to detect the factors that determine trusting the source of information, how it is presented, and its platform regarding skincare and cosmetic products.

## Materials and methods

Study design

This research employed a questionnaire-based cross-sectional study design to investigate the impact of social media on the choice of skincare and cosmetic products among adult females in Saudi Arabia. The study aimed to gather data from a diverse sample of participants to examine their social media usage patterns, sources of information related to skincare and cosmetic products, and the influence of social media on their decision-making process.

Participant selection

The target population comprised adult female residents across Saudi Arabia. To ensure the relevance and validity of the study findings, specific exclusion criteria were applied. Female residents residing outside Saudi Arabia were excluded due to potential variations in cultural and social factors. Additionally, individuals under 18 years old were excluded as the study focused on adult females. Participants with impaired hearing or vision, severe neurocognitive disorders, or mental conditions that hindered effective communication were also excluded. Participants who refused to participate were excluded from the study to maintain voluntary participation.

Data collection

Data collection was performed using electronic data collection through Google Forms, chosen for its ease of administration and widespread accessibility. Google Forms provided a user-friendly platform for participants to complete the questionnaire. Anonymity was ensured by not collecting any nominative information that could identify individual participants. The survey link was distributed through various social media platforms to reach a wide and diverse audience of potential participants. This approach facilitated the recruitment of large sample size and enabled the collection of comprehensive and representative data.

Questionnaire design

The questionnaire consisted of three sections, each addressing specific research objectives. The first section aimed to gather demographic information, including age, gender, marital status, occupational and educational level, and residency. This information allowed for the characterization of the study sample and the exploration of potential associations between these demographic variables and social media usage patterns related to skincare and cosmetic products. The second section comprised closed-ended questions designed to identify the most commonly used social media applications among participants, the sources of information they relied on regarding skincare and cosmetic products, and the level of trust placed in these sources. Participants were asked to rate their trust in various sources of information, including dermatologists, skin estheticians, other healthcare professionals, and family and friends. Additionally, participants were asked to assess the amount of information obtained from social media sources, how it was presented, and the level of trust in these sources. These questions aimed to capture participants' perceptions and attitudes toward social media content related to skincare and cosmetic products. The third section of the questionnaire focused on assessing the effects of social media on participants' decision-making processes when choosing skincare and cosmetic products. This section explored how social media influenced participants' opinions, preferences, and purchasing behaviors. By gathering insights into the impact of social media on decision-making, the study aimed to identify potential positive or negative consequences of social media usage in this context. The survey utilized a self-structured questionnaire. The validity of the questionnaire was evaluated, and satisfactory Cronbach's alpha values exceeding 0.7 were obtained for all sections.

Data analysis

Following data collection, the collected responses were carefully reviewed, revised, and coded for analysis. The statistical software IBM SPSS version 22 (IBM Corp., Armonk, NY) was utilized for data management and analysis. Descriptive analysis techniques, such as frequency and percent distribution, were employed to summarize the categorical variables, including participants' socio-demographic data, types and frequency of social media platform usage, and the impact of social media on choosing skincare and cosmetic products. Crosstabulation was employed to explore potential factors affecting female participants' trust in the provided information on skincare and cosmetic products. By examining the relationships between variables, such as the sources of information and participants' level of trust, the study aimed to uncover patterns and associations. The Pearson chi-square test and the exact probability test for small frequency distributions were used to test associations between variables and determine statistical significance. A two-tailed test was employed for all statistical analyses, and a p-value less than 0.05 was considered statistically significant, indicating a meaningful relationship between the variables under investigation.

## Results

The study questionnaire was completed by a total of 1,174 females who met the inclusion criteria, with 33.1% from the northern region, 22.7% from the western region, 17.7% from the southern region, 13.9% from the eastern region, and 12.6% from the central region. The participants' ages ranged from 18 to over 40 years old, with a mean age of 22.5 ± 13.9 years. Out of the total, 655 (55.8%) were single, and 463 (39.4%) were married. Among the participants, 566 (48.2%) were students, 288 (24.5%) were employed, and 320 (27.3%) were housewives. Regarding education, the majority (73.9%; 868) had a university level of education or higher (Table 1).

**Table 1:** Personal characteristics of study females, Saudi Arabia

Personal data	No	%
Region		
Central	148	12.6
Northern	389	33.1
Eastern	163	13.9
Western	266	22.7
Southern	208	17.7
Age in years		
18-20	204	17.4
20-25	479	40.8
25-30	135	11.5
30-35	97	8.3
35-40	88	7.5
> 40	171	14.6
Marital status		
Single	655	55.8
Married	463	39.4
Divorced / widow	56	4.8
Work		
Not working	320	27.3
Student	566	48.2
Working	288	24.5
Educational level		
Below university	306	26.1
University / above	868	73.9

Among Saudi females in the study, the commonly used social media platforms included Snapchat (39.4%), TikTok (26.7%), Instagram (19.6%), Twitter (5.4%), YouTube (5.3%), and WhatsApp (2.6%). A total of 881 (75%) of the participating females reported using social media for more than an hour a day, 222 (18.9%) used it once or twice daily, only 45 (3.8%) used social media platforms once every few days, and 26 (2.2%) used it once per week (Table 2).

**Table 2:** Types and frequency of used social media platforms among Saudi females

Social media use	No	%
The most used social media platform		
Snap Chat	462	39.4
Tiktok	313	26.7
Instagram	230	19.6
Twitter	63	5.4
YouTube	62	5.3
WhatsApp	30	2.6
Others	14	1.2
How often do you use social media		
More than an hour a day	881	75.0
Once/twice a day	222	18.9
Once every few days	45	3.8
Once a week.	26	2.2

Among the female participants in the study from Saudi Arabia, a precise total of 51% became acquainted with skincare products through social media platforms. Regarding the sources of information about cosmetic and skincare products on social media, 33.2% mentioned dermatologists, 26.8% mentioned friends/family, 20.1% mentioned social influencers, 14.4% mentioned cosmetologists, and 5.5% mentioned healthcare staff. When it came to the information they obtained, 31.5% reported having a significant amount of information or even too much information about cosmetic and skincare products from these sources, while 43.4% had an intermediate amount of information. A total of 95.4% expressed their trust in the information obtained from these sources, with 44.9% having an intermediate level of trust, 19.6% having a high level of trust, and 6.4% having an excessive level of trust. Additionally, 91.3% of the female participants' confidence in the information about cosmetic and skincare products was influenced by the visual presentation, with 37.1% experiencing an intermediate level of influence, 22.8% experiencing a significant influence, and 10.2% experiencing an excessive influence. Precisely 73.5% of the participants stated that they had seen the latest advertisement for skincare products on social media. Among them, 30.8% were not entirely convinced, 13.8% expressed disbelief in advertising, while 28.9% were convinced and planned to purchase the product in the future, and 14.2% already had the product at home. Furthermore, 49.6% confirmed that they had purchased a product recommended by social media bloggers, 15.9% stated that they had never done so, and 28.4% were unsure if they had or not (Table 3).

**Table 3:** Impact of social media on choosing skin care and cosmetic products among females in Saudi Arabia

Effect of social media		No	%
How did you become familiar with skin care products?	Social media platforms	599	51.0
	Recommended by friends	242	20.6
	Physicians	232	19.8
	In a makeup store.	88	7.5
	Internet websites	5	0.4
	Others	8	0.7
The source of your information related to cosmetic and skin care products on social media?	Dermatologist	390	33.2
	Friends and family	315	26.8
	Social influencer	236	20.1
	Cosmetologist	169	14.4
	Health care staff	64	5.5
The amount of information related to cosmetic and skin care products that you have collected from this source	None	53	4.5
	Little	243	20.7
	Intermediate	509	43.4
	Much	251	21.4
	Too much	118	10.1
How much do you trust the information from these sources?	No trust	54	4.6
	Little	288	24.5
	Intermediate	527	44.9
	Much	230	19.6
	Too much	75	6.4
To what extent does visual presentation affect your confidence in information related to cosmetic and skin care products?	No effect	102	8.7
	Little	248	21.1
	Intermediate	436	37.1
	Much	268	22.8
	Too much	120	10.2
Where did you see the last advertisement for skincare products?	Social media	863	73.5
	Online (banner, advertisement)	179	15.2
	Newspaper / magazine	23	2.0
	Mass media (TV, Radio)	44	3.7
	I haven't noticed any ads recently	65	5.5
Did this ad convince you?	Yes, and I have the product at home.	158	14.2
	Yes, I will buy it in the future.	321	28.9
	Incredible advertising to me.	153	13.8
	I'm not really convinced.	342	30.8
	I don't know.	135	12.2
Have you ever purchased a product that social media bloggers told you about?	Yes, sure.	582	49.6
	Yes, Not consciously.	71	6.0
	Maybe, I'm not sure.	334	28.4
	Never.	187	15.9

Females' trust in the information provided on skincare and cosmetic products was influenced by various factors. Among university graduates, high trust was reported by 72.2% compared to 67% among others, and this difference was statistically significant (p = 0.049). Furthermore, 94.9% of females who had an excessive amount of information displayed high trust, while 90% of those with a substantial amount of information and 24.5% of those with very little information showed high trust as well (p = 0.001). Similarly, high trust was observed in 81.3% of females who obtained information from healthcare staff, compared to 76.4% for dermatologists, 74.6% for cosmetologists, and 63.2% for friends (p = 0.001) (Table 4).

**Table 4:** Factors affecting females trust regarding provided information of skin care and cosmetic products P: Pearson X2 test, $: Exact probability test *P < 0.05 (significant)

Factors	Social media information trust level				p-value
	No / little trust		Higher trust		
	No	%	No	%	
Age in years					0.376
< 20	63	30.9	141	69.1	
20-30	168	27.4	446	72.6	
> 30	111	31.2	245	68.8	
Marital status					0.252
Single	178	27.2	477	72.8	
Married	146	31.5	317	68.5	
Divorced / widow	18	32.1	38	67.9	
Work					0.923
Not working	96	30.0	224	70.0	
Student	163	28.8	403	71.2	
Working	83	28.8	205	71.2	
Educational level					0.049*
Below university	101	33.0	205	67.0	
University / above	241	27.8	627	72.2	
The most used social media platform					0.131^$^
Instagram	53	23.0	177	77.0	
Snap Chat	136	29.4	326	70.6	
Tiktok	103	32.9	210	67.1	
Twitter	22	34.9	41	65.1	
YouTube	14	22.6	48	77.4	
WhatsApp	11	36.7	19	63.3	
Others	3	21.4	11	78.6	
The amount of information related to cosmetic and skin care products					0.001*^$^
Little	143	58.8	100	41.2	
Intermediate	128	25.1	381	74.9	
Much	25	10.0	226	90.0	
Too much	6	5.1	112	94.9	
The source of your information related to cosmetic and skin care products on social media?					0.001*
Cosmetologist	43	25.4	126	74.6	
Dermatologist	92	23.6	298	76.4	
Friends and family	116	36.8	199	63.2	
Social influencer	79	33.5	157	66.5	
Health care staff	12	18.8	52	81.3	

Various factors were found to be associated with females' influence on social media when it came to purchasing skincare and cosmetic products in Saudi Arabia. The demographic groups that were most influenced included middle-aged females (aged 20-30 years; 88.1%), single females (85.6%), students (87.5%), and those with a lower secondary level of education (84.6%). In terms of the social media platforms that had the most influence, YouTube users were highly influenced (87.1%), followed by TikTok (86.9%), Snapchat (83.5%), Twitter (82.5%), Instagram (82.2%), and WhatsApp (76.7%) (Table 5).

**Table 5:** Factors associated with females influence by social media for purchasing skin care and cosmetic products, Saudi Arabia P: Pearson X2 test​​​ ​*P < 0.05 (significant)

Factors	Influenced by social media				P-value
	Yes		No		
	No	%	No	%	
Age in years					0.001*
< 20	172	84.3	32	15.7	
20-30	541	88.1	73	11.9	
> 30	274	77.0	82	23.0	
Marital status					0.252
Single	561	85.6	94	14.4	
Married	380	82.1	83	17.9	
Divorced / widow	46	82.1	10	17.9	
Work					0.009*
Not working	258	80.6	62	19.4	
Student	495	87.5	71	12.5	
Working	234	81.3	54	18.8	
Educational level					0.752
Below university	259	84.6	47	15.4	
University / above	728	83.9	140	16.1	
The most used social media platform					0.588
Instagram	189	82.2	41	17.8	
Snap Chat	386	83.5	76	16.5	
Tiktok	272	86.9	41	13.1	
Twitter	52	82.5	11	17.5	
YouTube	54	87.1	8	12.9	
WhatsApp	23	76.7	7	23.3	
Others	11	78.6	3	21.4	

## Discussion

Social media has changed the way many industries do business, and cosmetic marketing is no exception, which is known to be significantly influenced by promoting products on social media platforms. With the rise of platforms like Instagram and YouTube, companies can now directly target their audiences and promote their products in new and innovative ways [12,13]. Social media has revolutionized the advertising industry in numerous ways. One of the most significant impacts it has always had is on the cosmetics sector and how cosmetics are marketed by providing a platform for beauty influencers and makeup artists to showcase their skills and advertise products. In the past, cosmetics advertising was limited to prints and television; now, social media allows for more creativity in advertising and a wider audience reach [14,15]. The current study aimed to assess the frequency and impact of social media use on choosing skincare and cosmetic products among females using the most popular platforms in Saudi Arabia. The respondent distribution, which included 33.1% from the northern region, 22.7% from the western region, 17.7% from the southern region, 13.9% from the eastern region, and 12.6% from the central region, does not accurately reflect the actual population density in Saudi Arabia. Notably, the highest population densities are observed in the central region, followed by the western region. This discrepancy may be attributed to potential incomplete responses to the questionnaire.

Our study revealed that Snapchat (39.4%), TikTok (26.7%), Instagram (19.6%), Twitter (5.4%), YouTube (5.3%), WhatsApp (2.6%), and other applications (1.2%) were the most commonly used social media platforms, consistent with similar findings in previous literature [16-19], such as the studies by Bahkali et al. [16] and Alsuraihi et al. [17], found that Twitter, Facebook, and short message services were the most popular social media platforms among medical students in Saudi Arabia. It is essential to acknowledge that these studies [16,17], conducted in 2015 and 2016, with a primary focus on Twitter and Facebook platforms’ users, may have limitations in reflecting the present-day landscape of frequently used social media platforms or applications, thus necessitating further exploration using more recent data. Another study revealed that more than half of the Saudi population has been found to highly favor the use of WhatsApp and Twitter for gaining and exchanging knowledge [18]. Barlow et al.’s study showed that Facebook and YouTube were the most used platforms (99.4% and 96.9%) among medical students [19]. Nevertheless, it is pertinent to acknowledge that certain studies utilized for comparative purposes predate the establishment of TikTok in 2016, which has since gained significant prominence as one of the foremost social media platforms in terms of frequency of use. Our study also reported that out of the participants, about 75% use social media for more than an hour a day, 18.9% use it once to twice daily, while others use it once every few days (3.8%) and even once per week (2.2%). Concerning the impact of social media and its influence on purchasing skincare and cosmetic products, the current study showed that about half of the respondents became familiar with skin care products through social media platforms. As for sources of information related to cosmetic and skin care products on social media among the participants, one-third reported knowledge from dermatologists, more than one-fourth from friends and family, and one-fifth from social influencers. As for the amount of information gained, about one-third of the participants reported that they gained too much information related to cosmetic and skin care products with the help of social media. Most of the study participants reported that they trust information on social media and find it credible, and much of this confidence was found to be affected by the visual presentation of the promoted products. Our study also documented that about three-quarters of the subjects noted that they saw the last advertisement for skincare products on social media, confirming the power of advertisement and product promotion. One-fifth of participants were convinced and more than willing to buy the promoted products in the future, while less than half of the participants were either not convinced or did not believe in advertising. The study also revealed that about half of the subjects confirmed that they purchased a product that social media bloggers spoke about, and 15.9% reported that they never did, while 28.4% were not sure if they did or not. Similar findings regarding social media's influence on cosmetic purchasing were reported in various literature. Lee et al. [20] concluded that influencers have a personal predisposition to influence the purchasing decisions of other customers. Chapple et al. [21] also found that cosmetic and skincare industry influencers have a positive effect on consumers' purchase intentions, specifically for luxury brands. Another study concluded that the cosmetics industry has derived substantial benefits from the rise of social media, which is why beauty brands have increasingly turned to digital media to raise their visibility and credibility and boost their sales (Ridder, 2020). In addition, a consumer survey in 2019 revealed that consumers wanted to view more product pictures and videos when shopping online. This means that video displays are rapidly developing, and consumers are used to watching product videos to obtain more information about product appearance, and functions [22]. Bouhlel et al. [23] found that the credibility of a blog brings a positive attitude and helps promote products. Hsu and Tsou [24] and Yüksel [25] also supported that perceived credibility has a positive effect on purchase intention. In Saudi Arabia, Al-Yahya et al. [26] found that most of the subjects used social media for three to five hours daily, 24% used it for 6 to 10 hours daily, 18.4% used it for less than two hours, and only 10.5% used it for more than 10 hours daily. The use of social media, especially 15 minutes before sleep and 15 minutes after waking up, is highly linked to the decision-making of performing cosmetic surgeries. The study also assessed factors associated with females' influence by social media for purchasing skincare and cosmetic products, where the most influenced were middle-aged females (20-30 years; 88.1%), single females (85.6%), students (87.5%), and those with below secondary level of education (84.6%). As for influence by using social media, the most influenced were YouTube users (87.1%), TikTok (86.9%), Snapchat (83.5%), Twitter (82.5%), Instagram (82.2%), and WhatsApp (76.7%). Social media has transformed the cosmetics industry by providing a platform for beauty influencers and makeup artists to promote products, and the current study found that Snapchat, TikTok, and Instagram were the most commonly used platforms among participants in Saudi Arabia, with a significant portion of users spending more than an hour a day on social media, highlighting the potential impact of social media on purchasing decisions for skincare and cosmetic products; therefore, healthcare providers should leverage these platforms to provide accurate information, while individuals should critically evaluate social media content and seek information from reliable sources when making decisions about skincare and cosmetic products.

## Conclusions

In conclusion, our analysis showed that most study participants used social media for many hours daily, mainly Snapchat, TikTok, and Instagram. Also, social media was the main source of information regarding skin care products mainly dermatologists. More information available was associated with high trust and influence on females’ purchasing intention. YouTube, TikTok, and Snapchat were the most influential platforms on females’ intention toward using skin care products and cosmetics. It is recommended that it is important to understand that every skin is dissimilar, and our skincare needs differ from one another too. Taking a general opinion and following another individual’s choice of skincare products and cosmetic procedures might not give us the favorable consequences we look for. Ultimately, we all strive to look and feel beautiful, but relying entirely on social media, particularly for the care of our skin is not the best decision.
